# Risk of Abortion and Ectopic Pregnancy in Women with a History of Polycystic Ovary Syndrome: A Nationwide Population-Based Cohort Study

**DOI:** 10.3390/jcm14176325

**Published:** 2025-09-07

**Authors:** Jin-Sung Yuk, Sang-Hee Yoon, Seung-Woo Yang

**Affiliations:** 1 Department of Obstetrics and Gynecology, Sanggye Paik Hospital, School of Medicine, Inje University, Seoul 01757, Republic of Korea; 2 Research Institute of Medical Science, Konkuk University School of Medicine, Seoul 05029, Republic of Korea; 3 Sanford Consortium for Regenerative Medicine, School of Medicine, University of California, San Diego, CA 92093, USA

**Keywords:** polycystic ovary syndrome, abortion, ectopic pregnancy, hydatidiform mole

## Abstract

**Objectives:** The purpose of this retrospective cohort study was to ascertain the risk of abortion, ectopic pregnancy and hydatidiform mole development in women with polycystic ovary syndrome (PCOS) using data from Korea’s National Health Insurance Service. **Method:** The women aged 20–49 years who were diagnosed with PCOS between 1 January 2012 and 31 December 2020 were enrolled. The control group (non-PCOS group) was composed of women aged 20–49 years who visited medical institutions for health examinations during the same period. Women diagnosed with any cancer were excluded from both groups. Logistic regression analysis was used to evaluate the risks of abortion, ectopic pregnancy and hydatidiform mole in PCOS in the presence of certain pregnancy-related confounding factors. **Results:** A total of 724,307 women were extracted, 169,998 women without PCOS and 44,714 women with PCOS were enrolled in the study. The PCOS group had a higher incidence of GDM and endometriosis. Abortions, ectopic pregnancies and hydatidiform moles were higher in the PCOS group than in the control group (abortion: 14.7% vs. 7.3%, *p* < 0.001; ectopic pregnancy: 3.3% vs. 1.1%, *p* < 0.001; hydatidiform mole: 0.2% vs. 0.1%, *p* < 0.001). After adjusted logistic regression, PCOS was found to be a risk factor for abortion (RR = 1.473, 95% CI = 1.424–1.524; *p* < 0.001) and ectopic pregnancy (RR = 1.845, 95% CI = 1.716–1.984, *p* < 0.001) but not hydatidiform mole (RR = 1.225, 95% CI = 0.927–1.62, *p* = 0.154). **Conclusions:** A history of PCOS itself might increase the risk of abortion and ectopic pregnancy. These findings could be useful in prenatal counseling and the management of patients with PCOS-associated pregnancies.

## 1. Introduction

Polycystic ovary syndrome (PCOS) is the most common endocrine disorder in women of reproductive age, with an incidence rate between 5% and 15% [1]. PCOS is associated with an increased risk of metabolic complications such as diabetes, dyslipidemia, and obesity. In terms of fertility, women with PCOS are at increased risk of infertility related to anovulation. The leading symptoms may vary with age, and treatment may be tailored to specific individual needs [2].

The fundamental defect in most cases of PCOS is functional ovarian hyperandrogenism (FOH) due to intrinsic theca cell dysfunction [3]. Hyperinsulinemia, a common feature of PCOS, acts on theca cells to exacerbate hyperandrogenism. Excess androgen causes premature luteinization, which hinders ovulation by impairing the selection of the dominant follicle [4]. Thus, for women with PCOS who are trying to conceive, various methods are being explored to increase ovulation rates, such as weight reduction; treatment with letrozole, metformin, and gonadotropin; and IVF-ET [2]. Once pregnant, women with PCOS appear to have an increased risk of obstetrical complications such as gestational diabetes (GDM) and pregnancy-induced hypertension (PIH) [5]. There is sufficient evidence for an increased incidence of GDM and PIH in women with PCOS [6,7,8].

However, few studies have investigated the associations between PCOS and early pregnancy complications such as abortion, ectopic pregnancy and hydatidiform mole. Most of the limited research has involved a retrospective audit of patients undergoing IVF. Homburg et al. reported an association between PCOS and an increased incidence of abortion [9]. The largest meta-analysis suggested that PCOS, independent of obesity, increases the risk of an abortion [8,10]. However, the inherent limitations of meta-analyses include a notable heterogeneity in the included samples, mainly due to varying study designs and ethnic backgrounds.

One retrospective cohort study indicated that PCOS was associated with an increased risk of ectopic pregnancies after in vitro fertilization–fresh ET cycles. However, the sample size was relatively small [11]. Another meta-analysis on adverse pregnancy-related outcomes in women with PCOS undergoing IVF was reported in 2019. No significant difference in the ectopic pregnancy rate was detected between women with and without PCOS [12]. The studies described above were characterized by pregnancies achieved through IVF and included participants from diverse ethnic backgrounds.

Patients with PCOS exhibit significant differences in phenotypes depending on the home country and ethnicity of the patient. Therefore, the purpose of this retrospective cohort study was to ascertain the risk of abortion, ectopic pregnancy, and hydatidiform mole development in reproductive-aged women with PCOS by analyzing nationwide population-based data acquired from the Health Insurance Review and Assessment Service (HIRA) of Korea.

## 2. Materials and Methods

### 2.1. Database

A single-payer system covers the majority of Korea’s population. The National Health Insurance Service of the Republic of Korea (NHIS) provides health insurance services to all residents [13,14]. The NHIS database contains medical records (sex, age, diseases, prescription medicines, surgeries, income level, type of medical insurance, and hospitalizations). To prevent disagreements between the NHIS and medical institutions over insurance premium payments, the Health Insurance Review and Assessment Service (HIRA) assesses the reasonableness of medical bills. Consequently, the HIRA stores most of the NHIS’s medical record data. This retrospective population-based cohort study investigated HIRA health insurance data from 1 January 2011 through 31 December 2020.

### 2.2. Selection of Participants

The International Classification of Diseases, tenth revision (ICD-10) and Korea Health Insurance Medical Care Expenses (version 2016, 2019) were utilized to select and validate outcomes. The PCOS group consisted of 20- to 49-year-old women diagnosed with PCOS between 1 January 2012 and 31 December 2020. Only women who visited medical institutions three or more times with a PCOS diagnosis code (E28.2) were included in the PCOS group. In South Korea, the clinical guidelines of the Korean Society of Gynecology and Endocrinology suggest only the Rotterdam 2003 criteria for the diagnosis of PCOS [15]. For the PCOS group, the day of enrollment was defined as the patient’s first visit to a medical clinic for PCOS. The non-PCOS group consisted of 20- to 49-year-old women who visited medical facilities for health examinations between 1 January 2012 and 31 December 2020. Women with PCOS were excluded from the group of women without PCOS. If a medical facility was visited more than twice for a health examination, the date of the first visit was used as the enrollment date. As a washout measure, the study excluded women who visited the hospital for PCOS or a health check-up in 2011. Women diagnosed with any cancer (C.xx) within 180 days of the day of inclusion into the study were excluded from both the PCOS and non-PCOS groups. Women with no pregnancy history in the entire cohort were excluded from both groups. Additionally, women with a history of abortion before the inclusion date were excluded from both categories.

### 2.3. Outcome

Cases of abortion, ectopic pregnancy, and hydatidiform mole were defined as three or more visits to a medical institution with the diagnostic codes for abortion, ectopic pregnancy, and hydatidiform mole, respectively. In Korea, induced abortions are not covered by the National Health Insurance system and thus are not included in the claims database. Therefore, our dataset only captures abortions of natural origin. Specifically, we included cases coded as spontaneous abortion (O03), missed abortion (O02.1), and blighted ovum (O02.0) according to ICD-10 classification.

### 2.4. Variables

If the patient’s medical insurance type was classified as medical aid, the patient’s SES was classified as low. A patient was classified as a rural resident if the area of the hospital where the patient was treated was not an urban area. The Charlson Comorbidity Index (CCI) was calculated from health insurance information taken from one year before the study participation date to the study participation date [16]. The delivery record in the health insurance data was used to determine parity (primipara or multipara) and the occurrence of multiple pregnancies. The presence of hypertension (I10~I15), diabetes (E10~E14) and obesity (E66) was determined by two or more visits to a medical clinic with the corresponding diagnosis code. Adnexal surgery (e.g., excision of the adnexal tumor, adhesional adnexectomy, ovarian wedge resection, or incision & draining of the ovarian cyst) was considered if it was performed at least once before enrollment day. Before the inclusion date, persons who visited a medical facility at least twice with a diagnosis code for uterine fibroids (D25) or endometriosis (N80) were assumed to have the associated disease.

### 2.5. Statistics

The SAS Enterprise Guide 7.15 (SAS Institute Inc., Cary, NC, USA) was utilized for all statistical calculations, while R 3.5.1 (The R Foundation for Statistical Computing, Vienna, Austria) served as a supplement. All the data were analyzed using a two-sided test, and a *p* value of 0.05 or less was regarded as statistically significant. Pearson’s chi-square test or Fisher’s exact test was used for categorical variables, whereas the *t* test or Mann–Whitney *U* test was used for continuous variables. The risk of abortion, ectopic pregnancy, and hydatidiform mole in patients with PCOS was determined using logistic regression analysis to account for confounding variables. The pairwise deletion method was employed when the missing value was less than 10%. However, the regression imputation method was utilized when the missing value was more significant than 10%. We performed logistic regression analysis to estimate the risks of abortion, ectopic pregnancy, and Hydatidiform mole development for PCOS in women with moderate to high SES to evaluate the validity of our study’s findings.

### 2.6. Ethics

The study was conducted in accordance with the Declaration of Helsinki and was approved by the SGPAIK IRB ethics committee (protocol code SGPAIK IRB 2021-08-001 and approval on 18 August 2021). In this study, variables that could be used to identify individuals were deidentified by the HIRA. The analysis for this study was performed on the HIRA-provided secure server, and only the outcome data (tables, figures, and numbers) are exportable from the server. Because the individuals cannot be identified, there is no risk to the study participants. Moreover, the Bioethics and Safety Act of South Korea does not require informed consent. Only the study results are exportable from the server under HIRA’s privacy policy. Thus, readers cannot access raw data. HIRA has no interest in this study despite this study’s use of HIRA data.

## 3. Results

**Figure 1:**
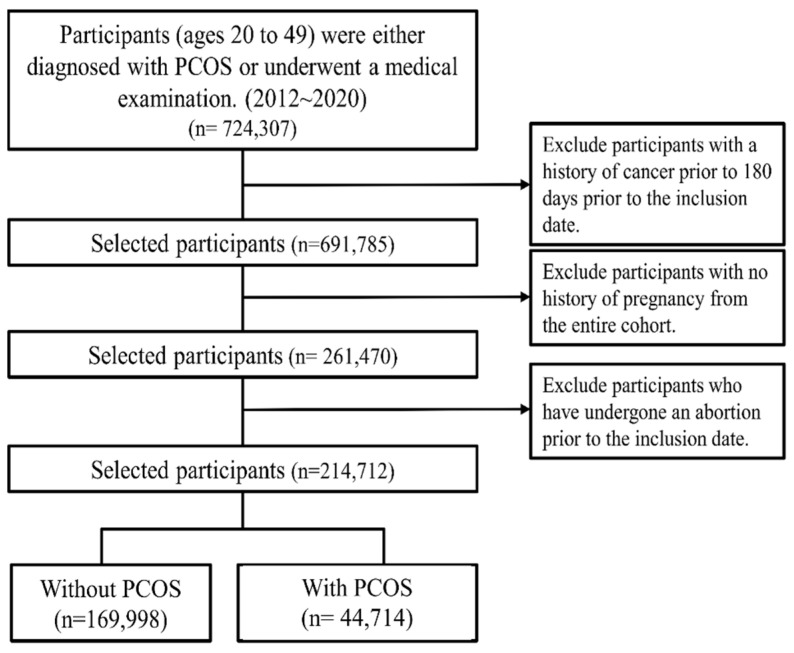
Flowchart for choosing participants for PCOS and non-PCOS.

The data from a total of 724,307 women (aged 20–49 years) who underwent a medical checkup or were diagnosed with PCOS in the HIRA database from 2012–2020 were included. Women under the age of 20 were excluded due to adolescence; the diagnosis of PCOS can be challenging because menstrual irregularity and hormonal imbalance during puberty may complicate the application of the Rotterdam criteria [17]. Women with no pregnancy history in the entire cohort were excluded from both groups. Additionally, women with a history of abortion before the inclusion date were excluded from both categories. Finally, 169,998 women without PCOS and 44,714 with PCOS were included (Figure 1).

Table 1 lists the detailed characteristics of the enrolled women. The rate of primiparity was greater in the PCOS group than in the non-PCOS group, and the median age was younger in the PCOS group than in the non-PCOS group. Additionally, pregnancy complications, such as GDM and endometriosis, were statistically higher in the PCOS group.

Table 2 indicates that the incidence rates of abortion, ectopic pregnancy and hydatidiform mole development were higher in the PCOS group than in the control group (abortion: 14.7% vs. 7.3%, *p* < 0.001; ectopic pregnancy: 3.3% vs. 1.1%, *p* < 0.001; hydatidiform mole: 0.2% vs. 0.1%, *p* < 0.001).

**Figure 2:**
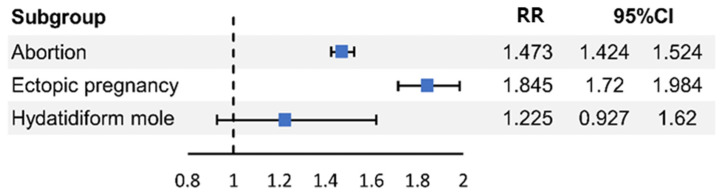
Relative risks of abortion, ectopic pregnancy, and hydatidiform mole in participants with and without PCOS.

In Table 3 and Figure 2, adjusted logistic regression was performed using SES, with the CCI and pregnancy complications as covariates. PCOS was a risk factor for abortion (RR = 1.473, 95% CI = 1.424–1.524, *p* < 0.001) and ectopic pregnancies (RR = 1.845, 95% CI = 1.716–1.984, *p* < 0.001) but not hydatidiform moles (RR = 1.225, 95% CI = 0.927–1.62, *p* = 0.154).

According to the subgroup analysis, PCOS was a risk factor for abortion across all age groups. For ectopic pregnancies, PCOS was a risk factor in relatively young women. No statistically significant association was found between PCOS and Hydatidiform moles (Table 4).

## 4. Discussion

In this study, PCOS was associated with an increased risk of abortion and ectopic pregnancy. This result suggests that PCOS is related to early implantation and the embryonic development process. In most cases, no apparent cause of early pregnancy loss can be identified; however, in addition to defects in the developing embryo, adverse alterations in endometrial function are likely to play a role [18]. In 1998, Okon, Laird et al. reported that women with recurrent abortion exhibit an abnormal decrease in the production of endometrial proteins such as PP14 during the secretory phase of the menstrual cycle, which appears to play important roles in endometrial receptivity during implantation [19].

The mechanism by which insulin resistance contributes to abortion is unclear, but it may involve the suppression of glycodelin and insulin-like growth factor-binding protein-1 (IGFBP-1) production in the endometrium, leading to an unfavorable environment for implantation. During the first trimester of pregnancy, the serum concentrations of glycodelin and IGFBP-1 are markedly reduced in women with PCOS. Glycodelin, produced by the endometrial glands during the luteal phase, inhibits the immune response of the endometrium to the embryo. On the other hand, IGFBP-1 is a protein that facilitates adhesion processes at the feto–maternal interface during the peri-implantation period [18].

This pathophysiological background raises the possibility that PCOS is an independent risk factor for early pregnancy complications. As mentioned before, this topic needs to be explored in a large cohort with a single Asian ethnicity. With respect to confounding factors, the analyzed dataset excluded a previous history of abortion and was adjusted for the number of deliveries, multiple pregnancies, GDM, PIH, adnexal surgeries, endometriosis, uterine leiomyoma and obesity, which affected the identified risks. After adjusting for confounding factors, PCOS was identified as a risk factor for abortion and ectopic pregnancy, whereas its relationship with hydatidiform mole development remained unclear in the homogeneous East Asian population.

We found that the PCOS group had a higher risk of abortion than the non-PCOS group did (RR: 1.473, 95% confidence interval (CI) 1.424–1.524). This finding is similar to the results of a meta-analysis conducted by [20], where they reported that women with PCOS had a greater incidence of abortion (OR: 1.59, 95% CI: 1.11–2.28). Both studies revealed an increased risk of abortion independent of obesity. Additionally, we performed an age-stratified analysis and found that the risk of abortion in patients with PCOS was exacerbated by age: (1) 20–29 years, relative risk (RR) 1.379, 95% confidence interval (CI) 1.307–1.454; (2) 30–39 years, relative risk (RR) 1.535, 95% confidence interval (CI) 1.468–1.605; (3) 40–49 years, relative risk (RR) 3.152, 95% confidence interval (CI) 2.526–3.934. Interestingly, patients over 40 years of age had an almost 3-fold greater risk than those in their 20s or 30s did, although the risk of ectopic pregnancy was similar across ages. Previous studies suggested that the frequency of clinically recognized early pregnancy loss for women aged 20–30 years is 9–17%, and this rate increases sharply to 40% at 40 years of age [21,22]. In this study, the PCOS group was nearly 3 times higher (in their 40s), which suggests that PCOS might be associated with adverse outcomes in older pregnant women. Faletta et al. suggested that older individuals with PCOS have a greater metabolic burden than young individuals do, which affects this result [20].

In our study, the PCOS group presented a greater risk for ectopic pregnancy (RR 1.845, 95% CI 1.716–1.984) than did the non-PCOS group. Endometriosis is the major risk factor for ectopic pregnancy [23]. According to the adjusted regression analysis, PCOS was associated with a greater relative risk than endometriosis (RR 1.324, 95% CI 1.152–1.522). Regarding ectopic pregnancies, previous studies have concentrated on the obstetric complications of IVF and compared PCOS with other causes of infertility. Jing Wang et al. performed a retrospective cohort study on the association between PCOS and ectopic pregnancies after IVF. They concluded that women with PCOS had a 3.06-fold greater risk of ectopic pregnancy than did those without PCOS after controlled ovarian hyperstimulation (COH) in the fresh ET cycle but not in the cryo-thawed ET cycle (adjusted OR, 3.06; 95% CI, 1.34–6.96). A possible explanation is that women with PCOS appear to have a lower threshold of hyperphysiologic estradiol levels that deleteriously impact tubal function [11]. However, Sha T. et al. aimed to study whether pregnancy-related complications differ between patients with PCOS and those with other causes of infertility who have undergone IVF. Twenty-nine eligible studies were reviewed, which increased the statistical power. According to the meta-analysis, no significant difference in the ectopic pregnancy rate was detected between women with and without PCOS (OR 1.52, 95% CI 0.96–2.41) [12]. Therefore, further studies are needed to investigate the independent influence of PCOS on ectopic pregnancies by performing subgroup analysis based on the mode of conception.

Oxidative stress is increased in individuals with PCOS and is positively correlated with BMI and insulin levels. In relation to insulin resistance, induced metabolic abnormalities increase oxidative stress in women with PCOS [24]. Hydatidiform moles are known placental malformations that cause early abortion and account for 1 in 41 cases of early pregnancy loss [25]. At the hydatidiform mole implantation site, trophoblastic atypia is diffuse. An increase in the number of infiltrating inflammatory cells in the molar implantation site triggers oxidative stress [26]. Harma et al. reported that patients with complete hydatidiform moles are exposed to increased oxidative stress, which may play a role in the pathogenesis of the disease or may be secondary to the disease [27]. Oxidative stress could serve as a connection, but whether PCOS plays a causative, incidental, or secondary role in hydatidiform moles has never been studied. Our retrospective cohort study revealed no association between PCOS and hydatidiform mole development. There was no difference in the risk of hydatidiform moles (OR 1.225, 95% CI 0.927–1.62) but further study will be necessary.

A limitation of this study is its retrospective design using an administrative database, which relies on the accuracy and consistency of the individuals coding the data. Obesity is a major clinical presentation of PCOS and a significant risk factor for early pregnancy loss. First, whether the degree of obesity is specifically coincident with the timing of abortion, ectopic pregnancy, or hydatidiform mole formation is not clear. Second, in this study, the incidence of obesity was very low in the control group (0.1%) and the PCOS group (0.2%), which might be due to missing data or inaccurate data coding. Therefore, further evaluations with other BMI data need to be performed. However, Ryu et al. reported only a small difference in the obesity rates between normal and PCOS groups in a Korean population (14.4% vs. 15.7%), which was much smaller than that reported in the cohort study performed by Mills et al. (3.5% vs. 22.3%) [7,28]. Therefore, although further confirmation is necessary, it appears that obesity did not exert marked effects in the present cohort. Additionally, although fertility treatment (ovulation induction and/or in vitro fertilization) and obesity treatment drug use are major issues associated with PCOS, our study did not adjust for these factors. However, it is unclear whether body mass index (BMI) or the use of fertility treatments (ovulation induction and/or in vitro fertilization (IVF)) played a role in the higher rates observed [29,30]. The incidence of abortion remains higher in women with PCOS, irrespective of obesity-related drug use such as metformin [31]. Unmeasured confounding factors such as certain lifestyle or environmental factors (e.g., smoking, nutrition, physical activity) were not available in the dataset and thus could not be adjusted for. In generalizability, as the study is based on the Korean population, the findings may not be fully generalizable to populations with different ethnic or healthcare backgrounds.

Despite these limitations, the strength of the present study is its assessment of pregnancy risk factors related to PCOS in a large cohort with a single Asian ethnicity. We included 214,712 women who had undergone PCOS or medical examinations between 2012 and 2020 (PCOS, 44,714 women; non-PCOS, 169,998 women). Multiple confounding factors, such as a history of previous abortion or ectopic pregnancies, were excluded, and parity, multiple pregnancies, adnexal surgeries, uterine fibroids, endometriosis, and obesity were adjusted to identify the independent risk of PCOS in pregnancy. The incidence of PCOS varies according to the diagnostic criteria employed. PCOS is commonly diagnosed using three diagnostic criteria: the National Institutes of Health (NIH) criteria, the Rotterdam criteria, and the Androgen Excess Society criteria [32]. During the study period, the clinical guidelines of the Korean Society of Gynecologic Endocrinology suggested the use of only the Rotterdam 2003 criteria for diagnosing PCOS, so the methodological heterogeneity was also adjusted.

In conclusion, a history of PCOS itself might increase the risk of abortion and ectopic pregnancies, but its relationship with hydatidiform mole development remains unclear. Although further studies are needed, the present findings will be helpful in prenatal counseling and the management of women with PCOS.

## Figures and Tables

**Table 1:** The characteristics of both PCOS and non-PCOS participants in this study.

	Control Group	PCOS Group	Total	*p*
Number of participants	169,998	44,714	214,712	
Median age (years)	33 [30–37]	30 [27–32]	32 [29–36]	<0.001
Median follow-up period (days)	1377 [561–2254]	1534 [789–2430]	1408 [614–2292]	<0.001
Age at inclusion (years)				<0.001
20~24	9041 (5.3)	5762 (12.9)	14,803 (6.9)	
25~29	32,547 (19.1)	16,039 (35.9)	48,586 (22.6)	
30~34	62,052 (36.5)	17,363 (38.8)	79,415 (37)	
35~39	44,900 (26.4)	4924 (11)	49,824 (23.2)	
40~44	18,320 (10.8)	575 (1.3)	18,895 (8.8)	
45~49	3138 (1.8)	51 (0.1)	3189 (1.5)	
SES				<0.001
Mid~high SES	168,253 (99)	44,458 (99.4)	212,711 (99.1)	
Low SES	1745 (1)	256 (0.6)	2001 (0.9)	
Region				<0.001
Urban area	94,197 (55.4)	23,033 (51.5)	117,230 (54.6)	
Rural area	75,801 (44.6)	21,681 (48.5)	97,482 (45.4)	
CCI				<0.001
0	135,612 (79.8)	36,863 (82.4)	172,475 (80.3)	
1	23,934 (14.1)	5778 (12.9)	29,712 (13.8)	
≥2	10,452 (6.1)	2073 (4.6)	12,525 (5.8)	
Delivery				<0.001
Primi	77,074 (45.3)	37,561 (84)	114,635 (53.4)	
Multi	92,924 (54.7)	7153 (16)	100,077 (46.6)	
Multiple pregnancy				<0.001
Singleton	168,098 (98.9)	44,541 (99.6)	212,639 (99)	
Multiple	1900 (1.1)	173 (0.4)	2073 (1)	
GDM				<0.001
Absent	161,328 (94.9)	40,958 (91.6)	202,286 (94.2)	
Present	8670 (5.1)	3756 (8.4)	12,426 (5.8)	
PIH				<0.001
Absent	169,591 (99.8)	44,681 (99.9)	214,272 (99.8)	
Present	407 (0.2)	33 (0.1)	440 (0.2)	
Adnexal surgery				<0.001
Absent	164,843 (97)	43,297 (98.2)	208,140 (97.2)	
Present	5155 (3)	787 (1.8)	5942 (2.8)	
Uterine fibroids				<0.001
Absent	156,169 (91.9)	42,921 (96)	199,090 (92.7)	
Present	13,829 (8.1)	1793 (4)	15,622 (7.3)	
Endometriosis				<0.001
Absent	162,748 (97.5)	41,522 (92.9)	207,270 (96.5)	
Present	4250 (2.5)	3192 (7.1)	7442 (3.5)	
Obesity				0.718
Absent	169,575 (99.8)	44,607 (99.8)	214,182 (99.8)	
Present	423 (0.2)	107 (0.2)	530 (0.2)	

CCI, Charlson comorbidity index; GDM, gestational diabetes mellitus; PIH, pregnancy-induced hypertension; PCOS, polycystic ovary syndrome; SES, socioeconomic status. Data are expressed as the number (%) or median [25th percentile, 75th percentile].

**Table 2:** Incidence rates of abortion, ectopic pregnancy, and hydatidiform mole that occurred among participants who did and did not have PCOS.

	Non-PCOS	PCOS	Total	*p*
Number of participants	169,998	44,714	214,712	
Abortion				<0.001
Absent	157,522 (92.7)	38,137 (85.3)	195,659 (91.1)	
Present	12,476 (7.3)	6577 (14.7)	19,053 (8.9)	
Ectopic pregnancy				<0.001
Absent	168,122 (98.9)	43,249 (96.7)	211,371 (98.4)	
Present	1876 (1.1)	1465 (3.3)	3341 (1.6)	
Hydatidiform mole				<0.001
Absent	169,814 (99.9)	44,634 (99.8)	214,448 (99.9)	
Present	184 (0.1)	80 (0.2)	264 (0.1)	

Data are expressed as the number (%); PCOS, polycystic ovary syndrome.

**Table 3:** Relative risks of abortion, ectopic pregnancy, and hydatidiform mole in participants with and without PCOS.

	Abortion		Ectopic Pregnancy		Hydatidiform Mole	
	RR (95% CI) ^a^	*p*	RR (95% CI) ^a^	*p*	RR (95% CI) ^a^	*p*
Crude						
PCOS	2.177 (2.109–2.248)	<0.001	3.036 (2.833–3.253)	<0.001	1.654 (1.272–2.151)	<0.001
Adjusted ^a^						
PCOS	1.473 (1.424–1.524)	<0.001	1.845 (1.716–1.984)	<0.001	1.225 (0.927–1.62)	0.154
Age at inclusion (years)						
20~24	1		1		1	
25~29	1.249 (1.173–1.33)	<0.001	1.007 (0.894–1.134)	<0.001	0.566 (0.371–0.864)	0.005
30~34	1.571 (1.478–1.67)	<0.001	0.968 (0.861–1.09)	<0.001	0.706 (0.475–1.049)	0.143
35~39	1.677 (1.567–1.794)	<0.001	0.815 (0.706–0.941)	<0.001	0.594 (0.369–0.957)	0.024
40~44	1.274 (1.164–1.394)	<0.001	0.447 (0.349–0.574)	0.105	0.617 (0.326–1.167)	0.185
44~49	0.435 (0.337–0.56)	<0.001	0.084 (0.027–0.262)	<0.001	2.351 (1.154–4.788)	<0.001
SES						
Mid~high SES	1		1		1	
Low SES	0.913 (0.765–1.09)	0.314	1.129 (0.779–1.638)	0.522	0 (0-Infinite)	0.971
Region						
Urban area	1		1		1	
Rural area	0.952 (0.923–0.982)	0.002	1.108 (1.033–1.188)	0.004	1.011 (0.79–1.293)	0.931
CCI						
0	1		1		1	
1	1.057 (1.01–1.105)	0.957	1.083 (0.978–1.199)	0.568	1.245 (0.89–1.743)	0.63
≥2	1.12 (1.05–1.198)	0.011	1.257 (1.091–1.448)	0.011	1.28 (0.789–2.075)	0.586
Delivery						
Primi	1		1		1	
Multi	0.227 (0.217–0.237)	<0.001	0.212 (0.189–0.237)	<0.001	0.376 (0.275–0.516)	<0.001
Multiple pregnancy						
Singleton	1		1		1	
Multiple	0.413 (0.288–0.594)	<0.001	0.829 (0.392–1.754)	0.624	0 (0-Infinite)	0.975
GDM	0.904 (0.786–1.041)	0.16	0.992 (0.687–1.432)	0.964	0.245 (0.034–1.764)	0.163
PIH	1.202 (0.756–1.912)	0.437	0.997 (0.247–4.015)	0.996	0 (0-Infinite)	0.988
Adnexal surgery before inclusion	0.907 (0.817–1.007)	0.067	1.063 (0.846–1.336)	0.601	0.816 (0.348–1.916)	0.641
Uterine fibroids	1.093 (1.028–1.162)	0.004	0.984 (0.793–1.22)	0.88	1.644 (1.085–2.489)	0.019
Endometriosis	1 (0.912–1.096)	0.994	1.324 (1.152–1.522)	<0.001	0.912 (0.447–1.861)	0.8
Obesity	0.814 (0.576–1.149)	0.241	1.249 (0.643–2.428)	0.512	0 (0-Infinite)	0.986

CCI, Charlson comorbidity index; CI, confidence interval; GDM, gestational diabetes mellitus; RR, relative risk; PCOS, polycystic ovary syndrome; PIH, pregnancy-induced hypertension; SES, socioeconomic status. ^a^ PCOS, SES, region, CCI, delivery, multiple pregnancies, GDM, PIH, adnexal surgery, uterine fibroids, endometriosis, and obesity were all considered when adjusting the relative risks.

**Table 4:** The relative risks of abortion, ectopic pregnancy, and hydatidiform mole in participants with and without PCOS by age.

	20~29 Years ^a^		30~39 Years ^a^		40~49 Years ^a^	
	RR (95% CI) ^a^	*p*	RR (95% CI) ^a^	*p*	RR (95% CI) ^a^	*p*
Abortion						
No PCOS	1		1		1	
PCOS	1.379 (1.307–1.454)	<0.001	1.535 (1.468–1.605)	<0.001	3.152 (2.526–3.934)	<0.001
Ectopic pregnancy						
No PCOS	1		1		1	
PCOS	2.009 (1.808–2.233)	<0.001	1.767 (1.597–1.956)	<0.001	1.475 (0.673–3.232)	0.332
Hydatidiform mole						
No PCOS	1		1		1	
PCOS	1.228 (0.807–1.868)	0.338	1.358 (0.924–1.997)	0.12	1.287 (0.302–5.493)	0.733

CI, confidence interval; RR, relative risk; PCOS, polycystic ovary syndrome. ^a^ PCOS, SES, region, CCI, delivery, multiple pregnancies, GDM, PIH, adnexal surgery, uterine fibroids, endometriosis, and obesity were all considered when adjusting the relative risks.

## Data Availability

Due to the Health Insurance Review and Assessment Service (HIRA)’s privacy policy, only authorized researchers can access the data.
